# Study protocol: Accommodative effects on the musculoskeletal system

**DOI:** 10.1371/journal.pone.0289347

**Published:** 2023-07-28

**Authors:** Grzegorz Zieliński, Anna Matysik-Woźniak, Beata Pająk, Michał Ginszt, Robert Rejdak, Piotr Gawda

**Affiliations:** 1 Department of Sports Medicine, Medical University of Lublin, Lublin, Poland; 2 Department of General and Pediatric Ophthalmology, Medical University of Lublin, Lublin, Poland; 3 Interdisciplinary Scientific Group of Sports Medicine, Department of Sports Medicine, Medical University of Lublin, Lublin, Poland; 4 Department of Rehabilitation and Physiotherapy, Medical University of Lublin, Lublin, Poland; Opole University of Technology: Politechnika Opolska, POLAND

## Abstract

Accommodation is a phenomenon of the eyeball, which consists of adapting to view objects at different distances. The main aim of this study is to evaluate the effect of accommodations on the musculoskeletal system in myopic and emmetropic subjects. To date, the effect of accommodative paralysis on the musculoskeletal system has not been studied. The research hypothesis based on the current research reports is that accommodation affects the musculoskeletal system in myopic and emmetropic subjects. A smaller aim of the study is to evaluate the effect of unilateral accommodative palsy on the musculoskeletal system in myopic and emmetropic subjects. According to the authors of the previous reports, unilateral accommodative palsy will affect increased musculoskeletal asymmetry in myopic and emmetropic subjects. The surface electromyography (sEMG) of the musculoskeletal system will be performed by using the Noraxon Ultium DTS 8-K MR 3 myo Muscle Master Edition. Cycloftyal (10 mg/ml, eye drops, solution) will be used to paralyze accommodation. After the paralysis of unilateral accommodation, the test will be repeated. Both-sided accommodation will be paralyzed next, and the test will be repeated. The study may provide insight into the effects of accommodation on the musculoskeletal system, and it may also help in understanding the connections between these systems.

## Introduction

Accommodation is the phenomenon of the eyeball, which consists of adapting to view objects at different distances. The adjustment of the eyeball involves the proper selection of the focal length of the optical system of the eye so that a sharp image of the viewed object is formed on the retina. This is related to the activity of the ciliary muscle. During accommodation, the ciliary muscle thickens anteriorly and thins posteriorly [1]. A recent study (2022) notes that people with myopia had longer and thicker ciliary muscles than those with emmetropia [1]. It has been suggested that the ciliary muscle may be important in developing myopia [2, 3]. One suggestion for touting the influence of the ciliary muscle on the onset of myopia is that its tension may affect the tension of the choroid and, as a result, cause a change in the axial length of the eyeball [4, 5]. Another explanation could be that changes in the morphology of the ciliary muscle (its thickness) restrict the equal expansion of the globe, leading to lens thinning and lower power [6]. There are other considerations explaining the involvement of the ciliary muscle in myopia [5]. Myopia is one of the most common ophthalmological problems in the world, and the number of people with myopia is steadily increasing, expected to reach 4.8 billion by 2050 [7]. The high prevalence rates pose a serious public health challenge due to the refractive error [8].

It has been observed that the bioelectrical activity of the masticatory muscles and the cervical spine muscles is related to the length of the eyeball, the thickness of the choroid, and the retina [9, 10]. One explanation for this phenomenon may be a connection in the musculo-fascial pathway. The muscles involved in the accommodation (the ciliary muscle and, indirectly, the extraocular muscles) of the eye are connected by fascial structures. The choroid membrane, cornea, lens, and retina form fascial networks that connect to the optic nerve and the eyeball [11]. Tenon’s fascia can be divided into two main parts: one containing the external muscles of the eyeball and the other connective tissue containing the optic nerve and connecting intraocular structures. Tenon’s fascia connects first to the upper eyelid lever muscle and then to the circular muscle of the eye, part of the SMAS (superficial musculoaponeurotic system). This system further connects to the fasciae of the whole body [9, 11].

In addition, several studies have observed differences in muscle activity during open-eye and closed-eye testing in people with myopia [12–14]. Such differences were not seen in emmetropic subjects [12, 15]. So far, it has been hypothesized to be explained by the vestibulo-ocular reflex and the connection to the trigeminal nuclei, which indirectly respond to impulses transmitted from other cranial nerves [16, 17]. The innervation of the ciliary muscle corresponds to the cranial nerve III additionally innervates the iris sphincter muscle, the levator palpebrae superioris muscle, and the extraocular muscles (superior rectus, inferior rectus, medial rectus, and inferior oblique muscle) [18]. These muscles are also connected by a fascicular network and it can be suggested that the influence of the organ of sight on the muscular system (and the muscular system on the organ of sight) takes place as a combination of both pathways [9, 16]. It is not known whether the connection between the systems (organ of vision and musculo-fascial) is primary or secondary.

This proprietary study is designed to investigate the effects of accommodation on the musculoskeletal system. Five ocular mydriatics and cycloplegics are currently available [19]. Cyclopentolate was first synthesized in 1951 by Treves and Testa [20]. Cyclopentolate hydrochloride is a cholinolytic agent that blocks muscarinic acetylcholine receptors. Muscarinic acetylcholine receptors include a family of five G protein-coupled receptors [19]. All five subtypes of muscarinic receptors are present in the human eye, especially in the iris-ciliary body [21]. Cycloplegics, by its action, mainly affects M3 receptors in the ciliary muscle and iris [22, 23]. Cholinolytic agents can exhibit types of actions: paralysis of the iris circular muscle, resulting in pupil dilation; and paralysis of accommodation, resulting in loss of near vision acuity [24].

Due to the observed connections, the authors decided to create the design of this study. The main aim is to evaluate the effect of accommodation on the musculoskeletal system in myopic and emmetropic subjects. To date, the effect of accommodative paralysis on the musculoskeletal system has not been studied. The research hypothesis based on the studies in our previous reports is that accommodations affect the musculoskeletal system in myopic and emmetropic subjects.

A smaller aim of the study is to evaluate the effect of unilateral accommodative palsy on the musculoskeletal system in myopic and emmetropic subjects. According to the authors of the study, unilateral accommodative palsy will affect increased musculoskeletal asymmetry in myopic and emmetropic subjects.

## Materials and methods

The study will be conducted by the recommendations of the Declaration of Helsinki and with the approval of the Bioethics Committee of the Medical University of Lublin (no. KE-0254/259/12/2022). The subjects will be informed of the objectives of the study, and they will be aware of the possibility of opting out at any time. All subjects will give their written consent to the study.

People aged between 20 and 30 will be invited to participate in the study. They will be students at the Medical University of Lublin after didactic classes conducted by the Department of General and Pediatric Ophthalmology and the Department of Sports Medicine. The students will be informed about the possibility of voluntary participation in the study. The information leaflets will be distributed. In addition, recruitment will be conducted at the Students’ Scientific Association at the Department of General and Pediatric Ophthalmology and the Interdisciplinary Scientific Group of Sports Medicine. The recruitment process will consist of a verbal presentation of the study assumptions and the distribution of information leaflets during the meeting of the above-mentioned students’ scientific associations.

Authors will have access to information that could identify individual participants during data collection. After data collection, the data will be anonymized.

The age criteria were chosen due to the rapid development of temporomandibular disorders (TMDs) at this age [25]. The refractive defect should then be stabilized [26]. The planned start of the study is April 2023.

Any participant will be able to stop the study at any time during the experiment. The experiment will be terminated if the following symptoms are reported by the patient or observed by the researcher: fever, hallucinations, an unusual feeling of weakness, redness on the face, or a skin rash.

The sample size was calculated using G*Power 3.1 software [27]. The minimum sample size was estimated at 18 eyeballs of individuals, assuming that this would be enough to see differences at a difference between two independent means (t-test), with an α value of 0.05, a power value of 0.95, and an estimated mean effect size of 0.75. Since, at first, we will conduct the study without accommodative paralysis, then after unilateral paralysis (18 eyeballs with accommodative paralysis to 18 eyeballs without accommodative paralysis in one group), and then after bilateral paralysis, a total of 32 people are needed (18 in the test group and 18 in control group).

The subjects will be divided into two groups: those without any refractive defect with 20/20 visual acuity and those with axial myopia with the best corrected visual acuity at the 20/20 level. Visual acuity testing will be performed according to the gold standard diagnostic—the Early Treatment Diabetic Retinopathy Study (ETDRS) chart [28]. Myopia will be defined according to the recommendations of The International Myopia Institute as a condition in which the spherical equivalent refractive error of an eye is ≤ −0.5 D when ocular accommodation is relaxed [29].

The following exclusion criteria from the study will be applied:

- Temporomandibular disorders;
- Classes II and III occlusion according to Angle’s classification;
- Open bite;
- Lack of preserved four zones of support in the dental arches;
- Any inflammation within the oral cavity;
- Disease or injury in the cervical spine;
- Possession of orthodontic braces;
- Possession of dentures;
- Skin diseases of the head and neck region;
- Facial hair that prevents electromyographic examination of facial muscles;
- Neurological disorders in the head and neck area;
- Cancerous diseases (regardless of type and location);
- Injuries to the head and neck region within the last 6 months before the examination;
- History of surgical treatment in the head and neck region within the last 6 months before the examination;
- Presence of metal components in the head and neck area;
- Implanted electronic devices;
- Pregnancy;
- Breastfeeding women;
- Restrictions in the mobility of mandible, joints of the upper limb, lower limb, and spine;
- Current infections;
- Body temperature above 37.5°C;
- Constant intake of medications, regardless of type;
- Wearing contact lenses 48 hours before the examination;
- Hyperopia;
- Best-corrected visual acuity below 20/20;
- Diseases of anterior ocular structures, regardless of type, determined by slit lamp;
- Intraocular pressure higher than 22 mmHg, as determined by the TonoPen device [9];
- Hypersensitivity to the active substance (cyclopentolate hydrochloride) and excipients:
  ○ Boric acid;
  ○ Potassium chloride;
  ○ Disodium edetate;
  ○ Sodium carbonate;
  ○ Benzalkonium chloride;
  ○ Water for injection;
  ○ Sodium hydroxide 40% (to determine pH);
  ○ Hydrochloric acid 10% (to determine pH);
  ○ Sodium chloride.

If latent hypermetropia is detected after cycloplegia, or if subjects with mild myopia detect excess accommodation or contracture after cycloplegia, the study will be discontinued, and subjects will be excluded from the study. If there are any adverse effects (including confusion, hallucinations, fever, skin rash, etc.), the study will be stopped, and participants will be excluded from the study.

Inclusion criteria:

- Four spheres of support in the dental arch and full dentition;
- No refractive error or myopia;
- 20/20 visual acuity.

### Examination procedure

**Step A**—The surface electromyography (sEMG) of the musculoskeletal system will be performed using the Noraxon Ultium DTS 8-K MR 3 myo Muscle Master Edition. The skin of the test area will be cleaned with 90% ethanol to reduce impedance. The test will be conducted in the morning to minimize the influence of diurnal fluctuations on muscle activity. Before each signal recording, the impedance will be checked. The placement of surface electrodes will be carried out according to the guidelines of the *surface EMG for a non-invasive assessment of muscles (SENIAM)*—the surface electromyography program [30]. The recording of the sEMG signal will be carried out at rest and during motor tasks (isometric contraction). The maximum voluntary contraction will be induced according to the current recommendation [31]. The following surface electrodes will be used for the study: Ag/AgCl with a diameter of 30 mm and a conductive surface of 16 mm (SORIMEX, Torun, Poland).

The following muscles will be analyzed:

- The anterior part of the temporalis muscle (TA);
- The superficial part of the masseter muscle (MM);
- The middle part of the sternocleidomastoid muscle (SCM);
- The upper part of the trapezius muscle (UT);
- The upper part of the rectus abdominis muscle;
- The lower part of the rectus abdominis muscle;
- Biceps brachii muscle;
- Abdominal external oblique muscle.

The TA, MM, SCM, and UT muscles were selected for the study due to two factors: previous observations of changes in their activity in myopic subjects [10, 9, 13, 16, 32] and their possible influence on tension-type headaches [33, 34]. Two abdominal muscles (rectus abdominis muscle and abdominal external oblique muscle) were chosen because of their important function in lumbar spine movements [35, 36]. The biceps brachii muscle has been selected as an important muscle for proper upper limb function [37, 38].

**Fig 1:**
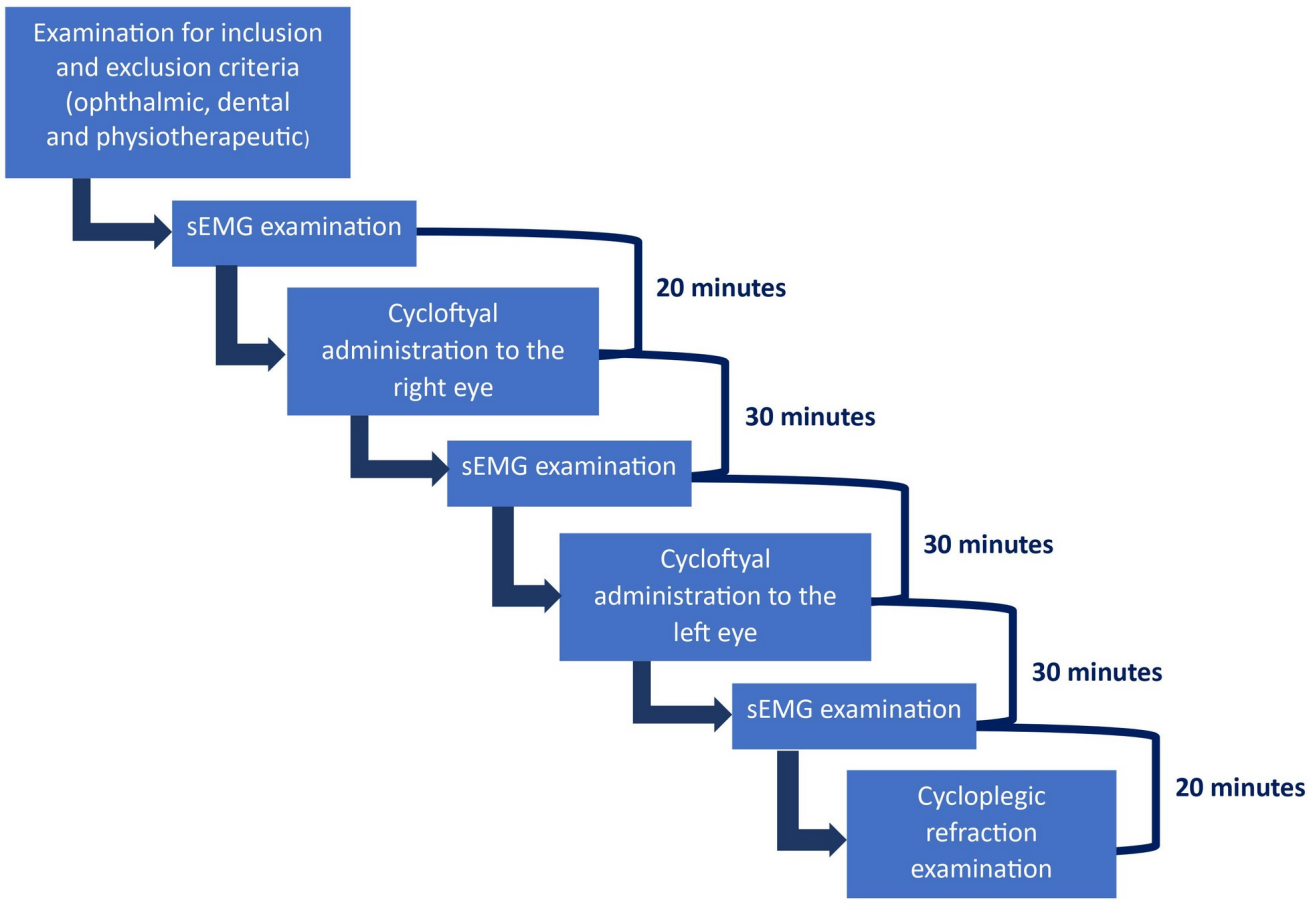
Course of the study.

The sampling frequency of the Noraxon Ultium sEMG sensors will be 2000 Hz. Noraxon MR3 software will be used to analyze the collected sEMG signals offline. This program will also be used for signal processing. First, a researcher specializing in electromyography (author G.Z.) will perform a visual analysis of the signal. Subsequently, standard processing of the sEMG kinesiology signal in the form of line cleaning and smoothing will be performed [31]. The test will be carried out at rest in a lying, sitting, and standing position. In addition, an isometric maximum voluntary contraction (MVC) test will be performed for each muscle group [39]. Items of MVC will follow international guidelines [31]. A 5-second maximum contraction will be performed, with a 2-second rest between, and then the contraction will be repeated [40]. Electromyographic activity will be recorded in conditions: at rest (10 s), during maximal voluntary contraction (as hard as possible; 3 × 3 s, 2 s rest between); in addition, one additional procedure will be applied for the masticatory muscles during maximal voluntary clenching on dental cotton rollers (as hard as possible; 3 × 3 s, 2 s rest between) [15, 41] (Fig 1).

**Step B**—Cyclopentolate hydrochloride (Cycloftyal, 10 mg/ml, eye drops, solution, manufacturer Verco, Warsaw, Poland) will be used to paralyze accommodation. One drop of Cycloftyal has a volume of about 0.03 ml, which means that one drop of the solution contains 0.3 mg of cyclopentolate hydrochloride. Cyclopentolate hydrochloride will be administered by an ophthalmologist. The medicinal product Cyclopentolate hydrochloride is indicated for topical use in the diagnosis of the fundus of the eye and refraction tests in adults, adolescents, and children over 3 months of age. It is also used as a pupil dilator in the treatment of iritis, iritis, and ciliary body inflammation, uveitis, and uveitis in adults [24, 42]. Cyclopentolate hydrochloride is a low-toxicity active substance with the advantage of inducing stable paralysis of accommodation. Maximum pupillary dilation after cyclopentolate administration occurs between 30 and 60 minutes of drug clearance. Complete resolution of pupillary dilation occurs after 24 hours. Maximum paralysis of accommodation after cyclopentolate administration occurs between 25 and 70 minutes after medication administration [24, 42]. In our study, patients will be given one drop of the solution. The dosage in all participants will be the same (Fig 1).

The procedure of the study will be as follows. First, a full sEMG will be performed after analysis of the inclusion and exclusion criteria. Then, the drug Cycloftyal will be administered to the right eye. After 30 min, the sEMG test will be repeated. Then, Cycloftyal will be administered to the left eye, and the sEMG will be repeated after 30 minutes (Fig 1).

### Statistical analysis

A statistical analysis will be carried out using Statistica™ version 13.3 (TIBCO Software Inc., Palo Alto, CA, USA). First, the normality of the distribution of the variables will be verified using the Shapiro–Wilk test and the Kolmogorov–Smirnov test (with Lillierfors correction). To compare two groups, when the distribution is close to normal, Student’s test will be used, while for independent samples, when the distribution deviates from normal, the Mann–Whitney U test will be used. For the analysis of three variables, the Analysis of Variance (ANOVA) test or the Kruskal–Wallis test will be used. Pearson’s test or the rho-Spearman test (for variables deviating from a normal distribution) will be used to analyze the correlation of variables. Differences will be considered statistically significant if the probability level of the test is lower than the assumed significance level (p < 0.05) and additionally (p<0.01) in the case of the rho-Spearman test. The effect size for the results obtained will be calculated [43]. The results will be presented graphically using GraphPad Prism 9 and LabPlot 2.9.0 (KDE, Berlin, Germany).

## Discussion

A failure of accommodation will result in the system’s inability to see objects at different distances. This will disrupt all three events that are responsible for changing the visual focus point: convergence of both eyes, contraction of the ciliary muscle, and constriction of the pupil [44, 45]. To date, the effect of accommodative paralysis on the musculoskeletal system has not been studied. Previous experiments conducted on people with myopia and people without myopia demonstrate the rapid response of the musculoskeletal system to visual responses. Changing the visual input from open eyes to closed eyes results in a change in the bioelectrical activity of muscles in people with myopia [12–14]. Moreover, responses of the muscular system were observed depending on visual acuity [32]. No changes in muscle activity were observed in emmetropic subjects depending on visual input [12, 15]. Based on these observations, we suggest that there will be an increase in resting activity and a decrease in the functional activity of the studied muscles after accommodative paralysis in both study groups. It has been observed that the bioelectrical activity of the muscles of the masticatory organ and the cervical segment is related to the length of the eyeball, the thickness of the choroid and the retina [9, 10]. According to the hypothesis, the tension of the ciliary muscle affects the tension of the choroid and changes the length of the eyeball [4, 5]. This may suggest that with unilateral accommodative paralysis, there will be a resting and functional decline on the side with paralysis and no change will be observed on the side without paralysis.

It is worth noting that the functioning of our body’s muscles is primarily determined by the nervous system. It initiates muscle activity by transmitting nerve impulses. In the case of the eye’s ciliary muscle, its activity is regulated by the parasympathetic nervous system. When the parasympathetic nerves innervating the ciliary muscle are stimulated, it contracts, leading to accommodation, which adjusts the focusing power of the eye. The activity of the studied muscles is mainly driven by nerve signals. Changing the activity of the ciliary muscle can affect other muscles through changes in nerve signals. The ciliary nerve (the third cranial nerve) is responsible for the activity of the ciliary muscle. Changes in stimulus perception through accommodative paralysis can affect other changes in neural conduction throughout the body. This may be related to reticular formation. The lateral parts of the reticular formation are close to various cranial nerves and influence their motor function [46]. The reticular activating system may also play an important role in modulating muscle tone [47, 48].

To date, the effect of vision on the muscles distal to the masticatory and cervical muscles has not been studied. The fascial continuum is involved in whole-body movements. Most skeletal muscles of the human body are directly connected by connective tissue [49]. Currently, in science, we can find several models explaining the transmission of forces through fascial tissue: the biotensegrity model, the fascintegrity model, and myofascial chains [50]. Regardless of the model, studies show that forces acting on specific muscle units can be transferred to other ones [50, 51]. Based on this, we surmise that, with unilateral as well as bilateral accommodative paralysis, there will be changes in all examined muscles.

The study may provide insight into the effects of accommodation on the musculoskeletal system. It may help in understanding the connections between anatomical systems.
